# Hints for the Sick-Room

**Published:** 1888-07-14

**Authors:** M. M. Basil

**Affiliations:** Author of "The Commoner Accidents to Life and Limb."


### HINTS FOR THE SICK-ROOM.

By M. M. Basil, M.A., M.B., C.M., Author of "The Commoner Accidents to Life and Limb."

### POULTICES, FOMENTATIONS, SINAPISMS, LOTIONS, Etc.—*(continued.)*

Perhaps the most convenient method of applying cold is by passing water of low temperature through coils of metallic tubes (Leiter's tubes) or indiarubber tubing. What is known as Thornton's ice-cap is a special form of such tubing. You must always adopt proper means to collect the moisture which is deposited on these tubes from the air in the form of dew. It may be well to remember that these tubes will also enable one to apply local heat very efficiently. Water of high temperature can be passed through them, provided there is a fold of flannel between them and the body. Some cases of very severe headache, as those of brain tumours, can be thus relieved when nearly everything else has failed. Cold may be applied conveniently to the head, especially in children, by wrapping two small ice-bags, each in a napkin, and pinning the napkin to the pillow, one on either side of the head.

In cases where dry cold applications are to be continued for a time it is best to interpose a thin covering between the skin and the ice, except in the scalp, where the hair acts as such protection. If, after getting over the first stage of nervousness, the patient complains of severe, persistent pain, report the fact to the doctor. When possible, make arrangements by which the weight of the ice-bag will, partially at least, be taken off an inflamed or painful part. A cradle placed over the portion of the body will enable you to suspend the bag to the frame so that it rests on the part with a fraction of its weight only. Cold may also be applied by what is known as "planing with ice." A slab of ice of convenient size is held in contact with the body under a sponge and moved about slowly, with an action of the arm imitating the process of planing wood: the sponge gets wet and must be squeezed dry from time to time.

The management of cooling or evaporating lotions need not receive further consideration. The essential condition of securing continual and free evaporation has been dwelt upon sufficiently. With a little ingenuity "drip-pots" containing the lotion may be suspended over the part. A leash of cotton thread or lamp-wick (perfectly free from any oil) may then be arranged to moisten the lint on the limb with a continuous but scanty supply of lotion.

Fomentations enable us to apply moist heat locally. Coarse flannel is the best material to use, as, owing to its interstices, it contains a large volume of air. It retains its heat for a long time, as air is a bad conductor of heat. The folds of flannel, which have been previously arranged to fit the area to be fomented, are dipped into boiling water on a towel or wringer, fished out on the towel, and wrung rapidly and completely dry. They are then applied to the surface, and covered with mackintosh or spongio-piline—not gutta-percha tissue or oil-silk. The mackintosh should completely overlap the flannel all round—not merely cover the surface of it. The whole work is a delusion if any the least portion of it is left unprotected. If the wringing is done thoroughly there need be no fear of scalding the skin by having the flannel too hot. Some authors insist a good deal on giving the folds of flannel a good shake before use, so as to imprison layers of air between them. A good shake will do no harm here, and you may give it on that ground. It is also a healthy exercise for the arms. It would, however, be interesting to understand what is supposed to occupy the space between the folds before shaking the flannel. Air must and will enter there, whether a shake is given or not. Fomentations may be kept on a quarter to half an hour or more, according to circumstances. A fresh one must be ready before removing the previous one.

### LEECHES, CUPPING, AND BLISTERS.

Leeches are seldom used in the practice of modern English medicine, and, judging from appearances, it does not seem likely that they will again come into fashion in the near future. As, however, in cases of eye diseases, ear affections, and ailments peculiar to children, they are sometimes employed; briefly, it is advisable to consider the essential rules which must be observed in their management.

Leeches must not be applied from patient to patient, otherwise diseases very disastrous in their results may be communicated by their agency. They must always be obtained from a respectable chemist. Leeches have some very remarkable oddities. It behoves us, therefore, to study their likes and dislikes with some care. They do not seem to appreciate any attention in the way of touching or holding between the fingers; grasping or squeezing them roughly is entirely out of the question. They will not bite a patient if he smells strongly of tobacco, or if the part offered to their attention is greasy or tastes salt, from want of scrupulous cleanliness. They are thorough believers in the use of cold water. They may be applied in various ways. Several of them may be placed in a small tumbler half full of cold water, the tumbler covered with a sheet of paper, inverted over the part and the paper withdrawn, whilst a sponge is applied against the side of the glass to soak up the water. Or you may have a test-tube containing some water capped over the leech, like the finger of a glove, from tail to head, not *vice versa*. The tube so charged is placed over the body and held in position till the animal bites. It is said that where they are over-pettish one may induce them to bite by smearing the bait with glycerine or syrup. It has also been suggested to cut an apple in two, scoop out one half, place the leech in the hollow so formed, and then apply it against the body.

It is important to bear in mind that a leech should not be pulled at or treated roughly after it once bites. Forcible pulling may divide the body from the head, and the teeth left in the flesh will cause considerable trouble in their extraction. Leeches can be induced to let go their hold with satisfactory despatch by putting a pinch of salt on them. A male patient can dislodge them quickly with a puff of tobacco smoke. The action of table salt on leeches is an important fact to bear in mind. You may have to apply leeches about the mouth, or in proximity to other cavities. If in such cases they crawl out of sight, salt water sent after them will render them harmless as far as any immediate risks are in question. In some countries—India, for example—leeches of several species abound in collections of fresh water. It is not uncommon to find that those who bathe in such water are considerably alarmed by having the animal lodged in the different cavities, such as the mouth, nose, etc. Salt water will, in all these conditions, act as an eloquent persuader to the intruders to let go their hold quickly.

The part of the body where leeches are to be applied will be selected by the doctor. In the absence of definite instructions on this point, the patient's friends should remember to select a spot where considerable pressure can be applied with a bandage in case of bleeding. This is best done by choosing such parts as are immediately over a bony surface. When leeches are to be applied to the head in children, it is best to choose the top of the head for the purpose. Bleeding can be stopped here easily. It has the further advantage that the wound will not annoy the patient by coming in contact with the pillows. Sometimes it is of particular advantage to apply them behind the ear.

*(To be continued.)*

